# Characterization and Phylogenetic Analysis of a Novel Uncultivated Magnetotactic Coccus Harbouring Multi‐Chain Magnetosomes

**DOI:** 10.1111/1758-2229.70266

**Published:** 2026-01-11

**Authors:** Yuzan Che, Wenyan Zhang, Yi Dong, Min Liu, Tian Xiao, Jin‐Yong Zhang, Hongmiao Pan

**Affiliations:** ^1^ The Laboratory of Aquatic Parasitology and Microbial Bioresources, School of Marine Science and Engineering Qingdao Agricultural University Qingdao China; ^2^ Laboratory of Marine Ecology and Environmental Sciences, Institute of Oceanology Chinese Academy of Sciences Qingdao China; ^3^ Laboratory for Marine Ecology and Environmental Science, Qingdao Marine Science and Technology Center Qingdao China; ^4^ Yazhou Bay Innovation Institute Hainan Tropical Ocean University Sanya China; ^5^ Laboratory for Marine Biology and Biotechnology, Pilot National Laboratory for Marine Science and Technology Qingdao China

**Keywords:** magnetosome gene cluster, magnetosomes, magnetotactic bacteria, magnetotactic cocci, polyphosphate

## Abstract

Magnetotactic bacteria (MTB) are a diverse group of microorganisms that synthesize intracellular magnetic nanocrystals termed magnetosomes. In this study, a novel marine magnetotactic coccus, designated strain HHB‐1, was magnetically enriched from intertidal sediments in Houhai Bay, southern China. Optical microscopy, electron microscopy and elemental analysis revealed that HHB‐1 cells are relatively large coccoid‐ovoid bacteria (3.9 ± 0.3 μm × 2.8 ± 0.2 μm) containing multiple chains of prismatic magnetite magnetosomes and prominent intracellular Ca/Mg‐rich polyphosphate (Ca‐Mg‐polyP) granules. Whole‐genome sequencing and phylogenomic analyses revealed that HHB‐1 represents a novel and deeply branching lineage within the order *Magnetococcales*, exhibiting low average amino acid identity (57.3%–58.7%) with previously reported strains. The magnetosome gene cluster (MGC) of HHB‐1 comprises a nearly complete set of *mam* (magnetosome membrane) genes with conserved gene order and structure, representing the first genomic and MGC characterization of a novel magnetococcus possessing multi‐chain magnetosomes. These findings expand our understanding of the diversity, biomineralization strategies and evolutionary history of MTB in marine environments.

## Graphical abstract

A novel marine magnetotactic coccus, strain HHB‐1, was isolated from intertidal sediments in Houhai Bay, China. HHB‐1 cells are large coccoid–ovoid bacteria that contain multiple chains of prismatic magnetite magnetosomes and conspicuous Ca/Mg‐rich polyphosphate granules. Genomic and phylogenomic analyses reveal that HHB‐1 represents a new deeply branching lineage within the Magnetococcales and harbours a nearly complete and conserved magnetosome gene cluster, providing the first genomic insights into a magnetococcus with multi‐chain magnetosomes.

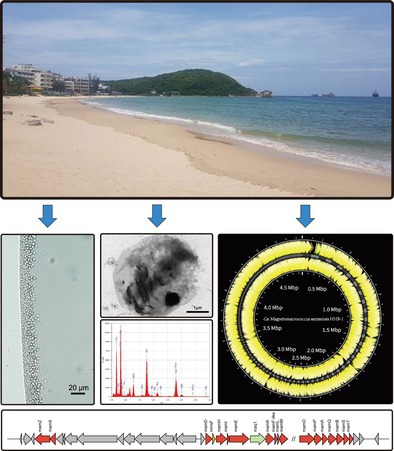

## 1 Introduction

Magnetotactic bacteria (MTB) are a group of widely distributed prokaryotes, recognized for their unique ability to sense and navigate along Earth's geomagnetic field. This capability is mediated by the intracellular magnetic nanoparticles, primarily magnetite (Fe_3_O_4_) and/or greigite (Fe_3_S_4_) enclosed in a lipid bilayer, known as magnetosomes (Balkwill et al. 1980). They are typically arranged in chains, thus forming a biological compass that enables MTB to efficiently migrate to their preferred niches at or just below the oxic‐anoxic interface (OAI) in aquatic environments (Frankel et al. 1997; Bazylinski and Frankel 2004). This behaviour is termed magneto‐aerotaxis (Frankel et al. 1997; Lefèvre et al. 2014). The biomineralization of magnetosomes (i.e., biosynthesis, arrangement, size and composition) is controlled by magnetosome gene clusters (MGCs), which are conserved genomic sequences within MTB genomes, containing a series of genes such as *mam*, *mms*, *mad*, *man* and *maq* (Jogler et al. 2009; Lin et al. 2017; Shimoshige et al. 2025). MTB have been discovered in both the Northern and Southern Hemispheres, widely distributed across various aquatic ecosystems, including lakes, rivers, brackish marshes and sediments. They play a significant role in global biogeochemical cycles, influencing the cycling of elements such as iron, carbon, nitrogen, sulphur and phosphorus through their unique biomineralization capabilities and metabolic activities. These functions make MTB an indispensable component of aquatic ecosystems (Lin et al. 2017).

MTB exhibit a diverse range of morphologies, including spirillum, coccus, vibrio, ovoid, rod‐shaped and multicellular forms. Taxonomically, according to the results of 16S rRNA gene sequences, genomes and the metagenome‐assembled genomes (MAGs) with MGCs, MTB are distributed in at least 17 phyla (Lin et al. 2020; Uzun et al. 2020; Wan et al. 2024). And those affiliated with the *Pseudomonadota*, *Nitrospirota and Desulfobacterota* (formerly known as *Proteobacteria*, *Nitrospirae and class delta‐proteobacteria*, respectively) have been the most extensively studied. Generally, magnetococci are the ubiquitous morphotype found in both marine and freshwater habitats (Liu, Liu, Zhao, et al. 2021). Initially, they were identified and classified within the *Alphaproteobacteria* class (Bazylinski et al. 2013). Recent integrative analyses combining genomic and metagenomic data from cultured and uncultured MTB have led to the proposal that magnetococci form a lineage, distinct from the *Alphaproteobacteria*, called the *Candidatus* Etaproteobacteria (Ji et al. 2017; Lin et al. 2018). Most recently, the Genome Taxonomy Database (GTDB) has redefined their taxonomic placement, assigning them to the *Magnetococcia* class (Sharma et al. 2022; Chuvochina et al. 2023). To date, although NCBI database queries reveal more than 1, 000 nucleotide sequences of 16S rRNA gene and 101 genomes affiliated within the *Magnetococcia* class, only six strains have been successfully isolated in pure culture. These cultivated representatives comprise marine‐derived strains *Magnetococcus marinus* MC‐1 (Bazylinski et al. 2013), *Ca*. Magnetococcus massalia MO‐1 (Lefèvre et al. 2009) and the unnamed isolate PR‐3 (Lefèvre et al. 2014); hypersaline lagoon‐adapted strains M*agnetofaba australis* IT‐1 (Morillo et al. 2014) and the unnamed isolate SS‐1 (Lefèvre et al. 2014); along with the most recently characterized freshwater isolate *Ca*. Magnetaquiglobus chichijimensis FCR‐1 (Shimoshige et al. 2025). With the exception of FCR‐1, which contains unchained magnetosomes, all others possess single‐chain magnetosomes. In addition, the majority of genomes within the *Magnetococcia* class originate from metagenome assemblies and lack corresponding morphological characterization, except for *Ca*. Magnetaquicoccus inordinatus UR‐1 which has descriptions of both MAG and morphological characteristics (Koziaeva et al. 2019). The freshwater strain UR‐1 exhibits unchained magnetosomes, sharing this morphological configuration with FCR‐1, despite their phylogenetic divergence within the *Magnetococcia* class.

Herein, we report a novel magnetococcus collected from intertidal zone sediments in Houhai Bay (Sanya city, southern China). Electron microscopy analysis and micromanipulation‐assisted single‐cell genome amplification revealed the first genomic/MGC characterization of a magnetotactic coccus strain possessing multi‐chain magnetosomes. Our findings contribute to the phylogenetic refinement of magnetococci and offer new insights into the biodiversity and phylogeny of MTB.

## 2 Materials and Methods

### 2.1 Sampling Site, MTB Collection and Optical Microscopy

In September 2024, sediments were collected from Houhai Bay (18°16′21″ N, 109°43′36″E) in Sanya city, located at the southernmost part of Hainan Island. The sediments were placed in 500 mL plastic bottles at a sediments:water ratio of approximately 1:1, transported to the laboratory and kept at room temperature in the dark for subsequent analysis. The sediment came from an environment with a salinity of ∼29.7 ppt, a pH of 8.2 and a temperature of 28°C. MTB cells were enriched by attaching permanent magnets to the outside of each plastic sampling bottle on opposite sides near the water/sediment interface (Pan et al. 2008). After 30 min, approximately 1 mL of water was drawn from the area near the magnets with a Pasteur pipette and transferred to centrifuge tubes for optical microscopy.

The morphology and motility of MTB cells were examined using an Olympus BX51 microscope equipped with differential interference contrast (DIC), phase contrast, fluorescence and a DP80 digital camera system (Olympus, Tokyo, Japan). MTB cells were observed in hanging drop within an artificial magnetic field (~25.5 G). A trajectory map of MTB cells' movement was produced with ImageJ (https://imagej.net/ij/).

### 2.2 Electron Microscopy and Energy Dispersive X‐Ray Spectrometry

Living cells, which were magnetically enriched as described above, were further purified using the ‘racetrack’ method as previously reported (Wolfe et al. 1987). For transmission electron microscopy (TEM) analysis, 3 μL of enriched MTB cells were adsorbed onto 200‐mesh carbon‐coated copper grids (Beijing Zhong Jing Ke Yi Technology Co. Ltd.), which were washed three times with Milli‐Q water and observed using a Hitachi HT7700 TEM (Hitachi Ltd., Tokyo, Japan) at 80 kV. Cell diameter, particle number, crystal length (along the long axis) and width (perpendicular to the long axis) of magnetosomes were measured from TEM images using Adobe Photoshop (version 21.2.2), and statistical analyses were performed with Origin (version 9.0.0). Meanwhile, the MTB cells that bound to the copper grids were also examined using a field emission scanning electron microscope (FESEM, ZEISS Gemini SEM 500, ZEISS Ltd., Germany) operated at 15 kV and a field emission transmission electron microscope (FETEM, JEM‐F200 JEOL Ltd. Japan) operated at 200‐kV equipped with an energy‐dispersive X‐ray spectrometer (EDXS).

### 2.3 Single‐Cell Sorting and Whole Genome Amplification

MTB cells were purified and sorted using a micromanipulation system, which included a TransferMan ONM‐2D micromanipulator, a CellTram Oil manual hydraulic pressure control system (IM‐9B) and an Olympus IX51 microscope (Tokyo, Japan). Procedures for single‐cell sorting have been described previously (Chen et al. 2015). The sorted MTB cells were transferred into 3 μL PBS and treated with repeated freeze/thaw cycles. Whole‐genome amplification (WGA) of MTB was performed using the REPLI‐g Single Cell kit (Qiagen, Hilden, Germany) following the manufacturer's protocol.

### 2.4 16S rRNA Gene Sequencing Analysis

The 16S rRNA gene was amplified from the WGA product. PCR was performed using bacterial universal primers 27f (5′‐AGAGTTTGATCCTGGCTCAG‐3′) and 1492r (5′‐GGTTACCTTGTTACGACTT‐3′) as previously described (Lin et al. 2011). The PCR products purified using the TIANgel Midi Purification Kit (TIANGEN, Beijing, China) were cloned into the pMD18‐T vector (TaKaRa, Japan), which was transformed into competent *E. coli* DH5α cells (Takara, Japan). Twenty clones were randomly selected for sequencing by Sangon Biotech (Shanghai, China). The 16S rRNA gene sequences were aligned using CLUSTAL W software, and sequence identities were determined using the BioEdit alignment editor (version 7.0.5.3). A 97% sequence identity threshold was applied for the operational taxonomic unit (OTU) identification. Representative sequences of each OTU were analysed using the BLAST search programme (http://www.ncbi.nlm.nih.gov/BLAST/).

Reference MTB sequences for 16S rRNA gene were downloaded from the NCBI database. All related sequences were aligned using BioEdit Alignment Editor software, using the CLUSTAL W multiple method. Alignments were corrected and trimmed manually. And a phylogenetic tree was subsequently constructed using the neighbor‐joining method (Saitou and Nei 1987) in the MEGA software package (version 11.0.13). Bootstrap values were calculated with 1000 replicates. The Representative 16S rRNA gene sequence was submitted to the GenBank database under accession number PV608029.

### 2.5 Genome Sequencing and Assembly

Paired end 150 bp (PE150) libraries were constructed from the WGA product. The genome was sequenced by Novogene Biotech (Beijing, China). Clean reads were assembled into contigs using IDBA (version 1.1.3), with k‐mer sizes ranging from 27 to 255 in steps of 20 (Peng et al. 2012). The metaWRAP (version 1.2.1) pipeline was used for metagenome binning, refinement and reassembly using default parameters to select the genome (Uritskiy et al. 2018). The quality of the MTB genome was assessed using QUAST (version 5.0.2) (Gurevich et al. 2013), and genomic completeness and contamination were estimated using CheckM (version 1.0.12) (Parks et al. 2015). The draft genome sequence was deposited in the GenBank database under the following accession number: JBQGBW000000000.

### 2.6 Genome Phylogenetic Analysis and Comparative Genomic Analyses of Magnetosome Genes and Proteins

GTDB‐Tk (version 1.4.0) was used to identify 120 concatenated bacterial single‐copy marker genes from the genome. These genes were aligned and concatenated for tree construction (Chaumeil et al. 2020). A maximum‐likelihood phylogenetic tree was reconstructed using IQ‐TREE (version 2.2.2.7) with the LG + F + R4 model, and branch support values were calculated from 1000 ultrafast bootstrap replicates using UFBoot2 (Hoang et al. 2018; Minh et al. 2020). The tree was rooted using a single outgroup taxon, *Magnetospirillum gryphiswaldense* MSR‐1. The visualization of the phylogenetic tree was performed using the online tool iTOL (https://itol.embl.de/). The average amino acid identity (AAI) of genomes was calculated, then hierarchical clustering of taxa was constructed on AAI values using EzAAI pipeline (version1.2.3) (Kim et al. 2021).

Gene prediction and annotation for the MTB genome, along with those of six representative MTB (UR‐1, FCR‐1, PR‐3, IT‐1, MO‐1 and MC‐1), were performed using Prokka (version 1.14.6) (Seemann 2014). The identification, annotation and visualization of MGCs were performed using MAGcluster (Ji et al. 2022) with manual inspection. A maximum‐likelihood phylogenetic tree was constructed based on the concatenated amino acid sequences of 14 conserved magnetosome proteins (MamH, I, E, K, F‐like, L, M, O, P, A, Q, B, S and T), using IQ‐TREE with the WAG + F + G4 model, with 1000 bootstrap replicates.

## 3 Results

### 3.1 Identification of a Dominant Magnetotactic Coccus

**FIGURE 1:**
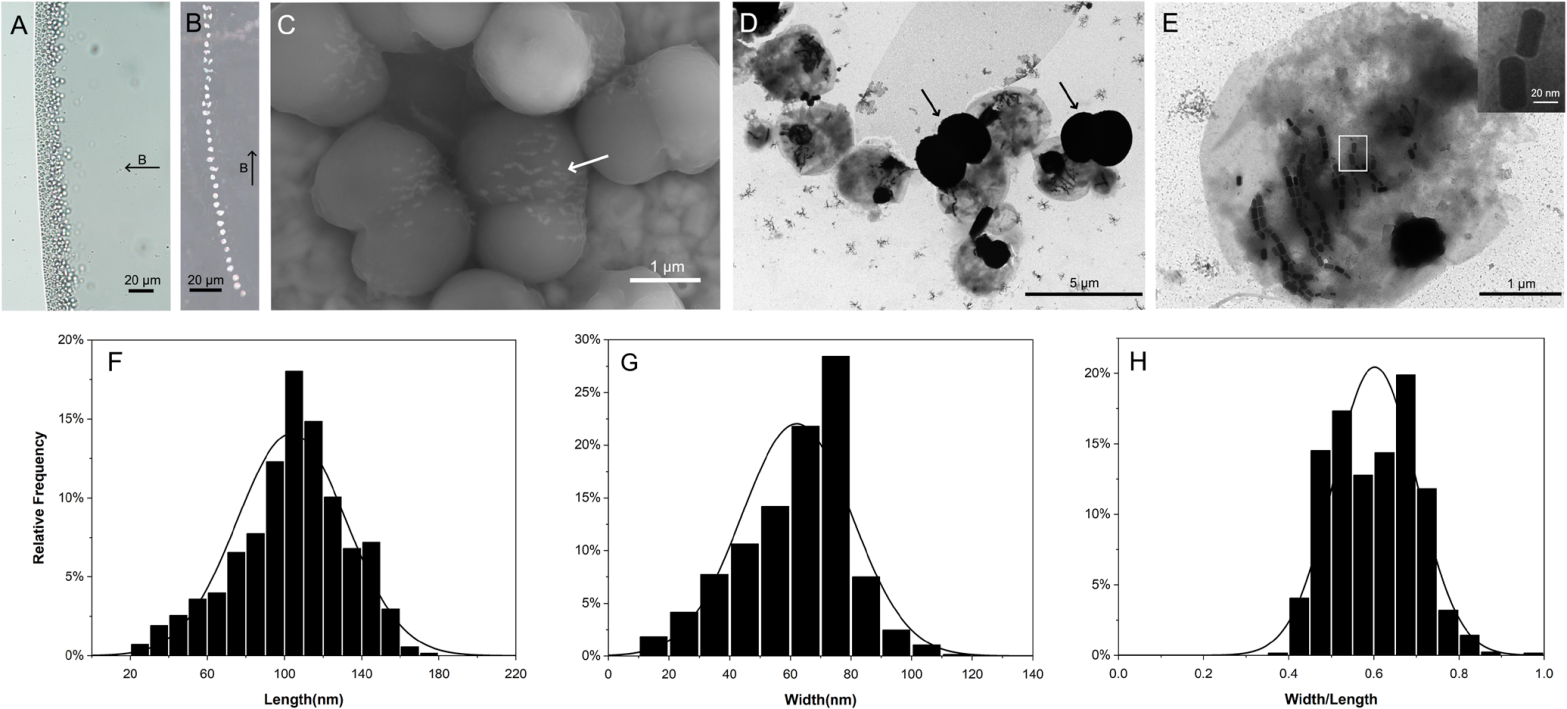
Morphological features of HHB‐1 cells. The HHB‐1 is a coccoid magnetotactic bacterial strain, isolated from the sediments of Houhai Bay and named after this sampling site (HHB: HouHai Bay). (A) Optical microscopy image of living HHB‐1 cells aggregated at the edge of the droplet. (B) Trajectory of an HHB‐1 cell swimming along the applied magnetic field. (C) Field emission scanning electron microscope (FESEM) image of HHB‐1 cells showing their morphological structures. The white arrows indicate the magnetosomes. (D) Transmission electron microscopy (TEM) image of HHB‐1 cells. (E) HHB‐1 cell from which black dense granules may have extruded. The inset in (E) shows a magnified image of the magnetosomes indicated by the white rectangle. (F–H) Frequency histograms of the length (F), width (G) and the width/length ratio (H) for prismatic magnetosomes in HHB‐1 cells (*n* = 1252). In panel A and B, the arrows labelled with B indicates the direction of the applied magnetic field (~25.5 G).

After a week of storage at room temperature, cocci were the predominant morphotype of MTB in sediment samples (up to 1.3 × 10^5^ cells/cm^3^) under optical microscopy (Figure 1A). Almost all cells were coccoid‐ovoid and had an average size of about 3.9 ± 0.3 μm by 2.8 ± 0.2 μm (*n* = 15) determined using bright‐field microscopy. Cells displaying this morphotype were selected through micromanipulation for subsequent phylogenetic analysis. The almost full‐length 16S rRNA genes of these cells were amplified, cloned and sequenced. A total of 20 nearly complete 16S rRNA gene sequences were obtained from 20 randomly selected clones, and all the sequences showed over 99.0% sequence identity to each other, forming a single OTU. This result provides evidence that this type of magnetotactic coccus functions as the ecological dominant MTB within the sampled microenvironment. These magnetococci cells are provisionally named after the site from which they were isolated: Houhai Bay (HHB)‐1.

### 3.2 Morphology, Magnetotaxis and Ultrastructure of HHB‐1 Cells

In a hanging drop, freshly collected magnetococci displayed north‐seeking magnetotaxis under an applied magnetic field, and swam towards the south pole with an average swimming speed of 39.48 ± 10.80 μm/s (*n* = 29) (Figure 1B). Under FESEM observation, the cells exhibited coccoid‐ovoid morphology, within which dumbbell‐like structures were discernible (Figure 1C), and multiple chains of magnetosomes were visible (indicated by white arrows in Figure 1C). TEM analysis of the magnetically enriched cells revealed that HHB‐1 cells were also coccoid‐ovoid shape, with an average diameter of 3.8 ± 0.4 μm × 2.7 ± 0.3 μm (*n* = 20) (Figure 1D). However, numerous dark, electron‐dense, dumbbell‐shaped granules were frequently observed adjacent to the cells (indicated by black arrows in Figure 1D). These extracellular granules resembled the dumbbell‐shaped structures in Figure 1C, suggesting a possible correspondence between the two. The magnetosomes were located on one side of the HHB‐1 cells and most of them were aligned into multiple chains (similar to the magnetosomes indicated by white arrows in Figure 1C), each generally containing no more than 10 magnetosomes, although a small portion were dispersed beside the chains (Figure 1E). On average, HHB‐1 contained 91 ± 17 prismatic magnetosomes per cell (*n* = 19). The magnetosomes exhibited average dimensions of 103.70 ± 28.33 nm in length (Figure 1F) and 62.15 ± 18.09 nm in width (Figure 1G), yielding a shape factor (width‐to‐length ratio) of 0.60 ± 0.10 (*n* = 1178) (Figure 1H).

### 3.3 Chemical Composition of a Single HHB‐1 Cell

**FIGURE 2:**
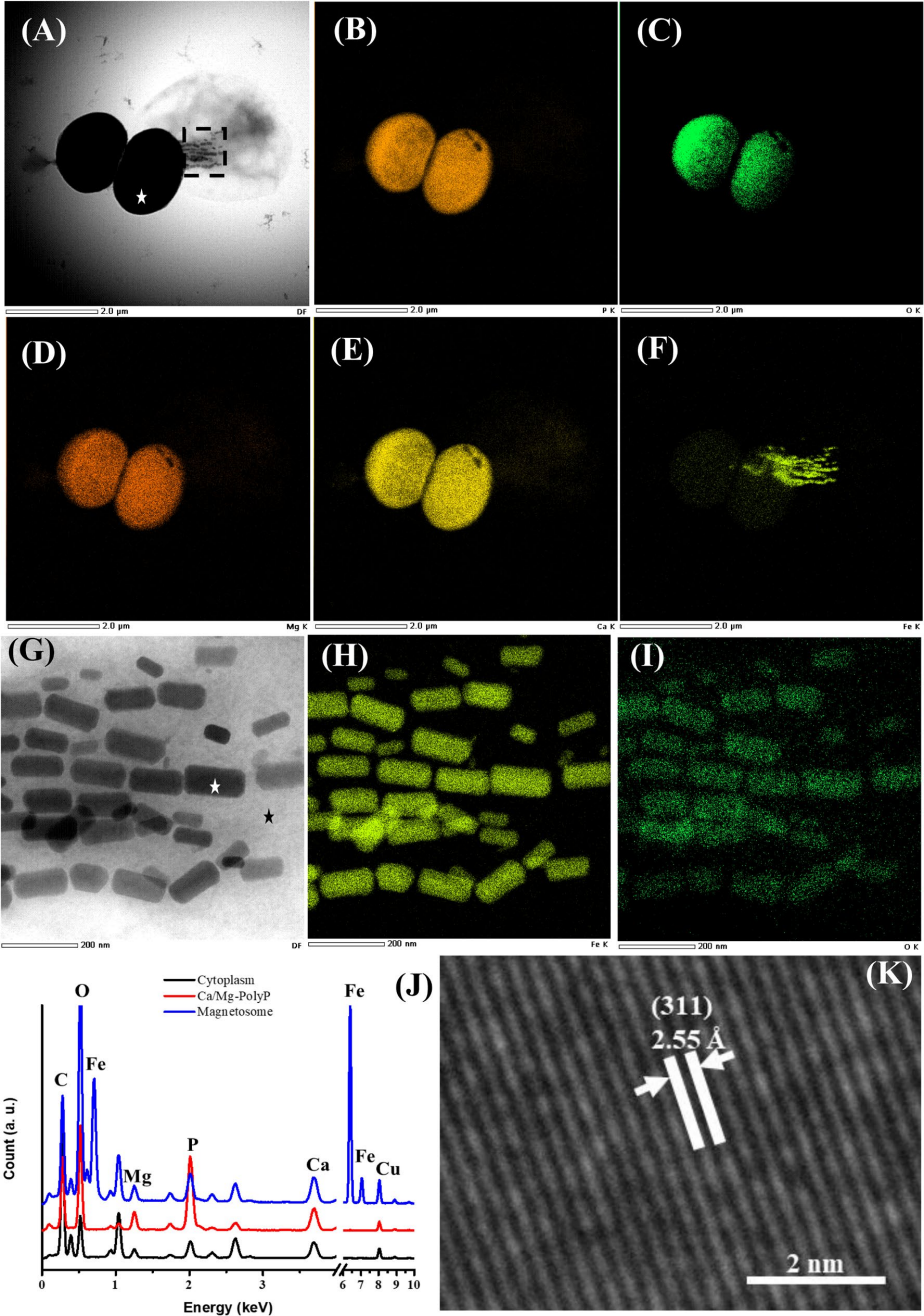
Major elements of the HHB‐1 cell and magnetosomes. (A) Dark Field (DF) image of HHB‐1 cell under FETEM. FETEM‐EDXS mapping of HHB‐1 cell, the corresponding chemical maps of phosphorus (P Kα) (B), Oxygen (O Kα) (C), magnesium (Mg Kα) (D), calcium (Ca Kα) (E) and iron (Fe Kα) (F). (G) DF image of magnetosomes (black rectangle in panel A) in the HHB‐1 cell under FETEM. FETEM‐EDXS mapping of HHB‐1 magnetosomes, the corresponding chemical maps of iron (Fe Kα) (H) and oxygen (O Kα) (I). (J) Energy‐dispersive X‐ray spectroscopy (EDXS) analysis of large electron‐dense particle, magnetosome and cytoplasm (corresponding to the sites denoted by star symbols in panels A and G). (K) High‐resolution transmission electron microscopy (HRTEM) image of a magnetosome (marked with white star symbol in panel G).

For elemental composition and distribution, EDX mapping was performed on an HHB‐1 cell which exhibited dark dumbbell‐shaped granules possibly released from its interior under FETEM (Figure 2A). EDX elemental mapping of whole cell revealed that O, P, Mg and Ca were primarily localized within the electron‐dense granule (Figure 2B–E), while Fe was specifically concentrated in the magnetosomes (Figure 2F). Therefore, the black electron‐dense particles observed in HHB‐1 cells can be designated as Ca/Mg‐rich polyphosphate (Ca‐Mg‐polyP) granules. Given that the characteristic spectral peaks of magnetosomes could potentially be obscured by signals from Ca‐Mg‐polyP granule, we specifically conducted independent EDX mapping on magnetosomes (Figure 2G, black rectangle in Figure 2A). In contrast, Fe and O specifically concentrated in the magnetosomes (Figure 2H,I). Subsequently, EDX point analyses were performed on the dark granule, magnetosome and cytoplasmic regions, yielding results consistent with those obtained from elemental mapping. Furthermore, the magnetosome lattice exhibited the characteristic d‐spacing of 2.55 Å corresponding to the (311) plane of magnetite, indicating that the magnetosomes were composed of Fe_3_O_4_, which is the common iron mineral formed by magnetococci.

### 3.4 Genome Assembly

Metagenomic sequencing of the WGA products resulted in 211 million reads, then these reads were quality trimmed and assembled into contigs. Binning of the contigs enabled reconstruction of a MAG of 4.96 Mb in size (Table S1). The MAG of HHB‐1 demonstrated 94.07% completeness with 7.14% contamination, meeting the medium‐quality criteria for draft genomes (Bowers et al. 2017). The genome contained 4348 protein‐coding sequences (CDSs), 48 transfer RNA (tRNA) genes and one 16S rRNA gene (Table S1). The 16S rRNA gene sequence exhibited 99.5–100.0% sequence identity with the amplified sequences described above.

### 3.5 Phylogenetic Analysis Based on Both 16S rRNA Gene Sequence and Metagenome‐Assembled Genome (MAG)

**FIGURE 3:**
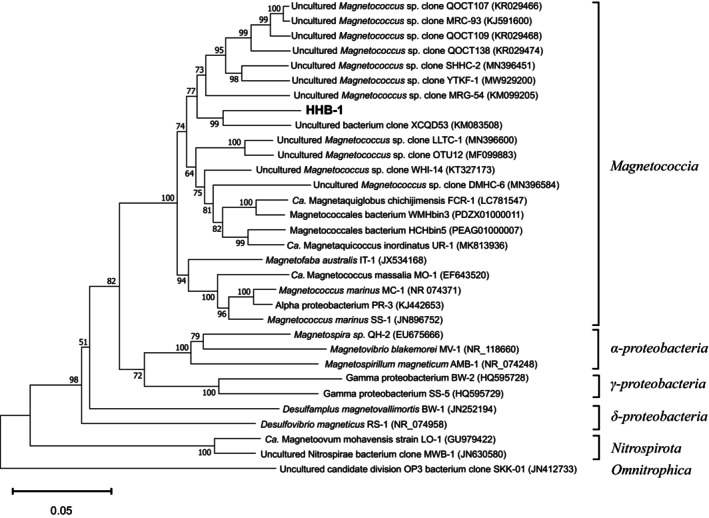
Phylogenetic tree based on 16S rRNA gene sequence of HHB‐1 and related MTBs, showing the position of strain HHB‐1 in the *Magnetococcia* class. Bootstrap values at the nodes are percentages of 1000 replicates. Bar represents 5% sequence divergence.

Among all 16S rRNA gene sequences in GenBank, the 16S rRNA gene sequence of HHB‐1 showed a maximum sequence identity (92.4%) with an uncultured bacterium clone XCQD53 collected from intertidal sediments (Taiping Bay, China; GenBank accession no. KM083508). Considering their 16S rRNA gene sequences had more than 7% divergence from all previously reported bacteria, they may represent a novel genus or family of Magnetococcia. From the phylogenetic tree of 16S rRNA gene, HHB‐1 grouped together with UR‐1, FCR‐1 and some uncultured magnetotactic cocci isolated from intertidal sediments, coral reef habitats, seamounts, freshwater lakes, freshwater rivers and estuarine regions. The clade was distinctly separated from the clade formed by several cultivated magnetotactic cocci, including *Mf*. australis IT‐1, *Ca*. Mc. massalia MO‐1, *Mc*. marinus MC‐1, SS‐1 and PR‐3. The phylogenetic analysis of the 16S rRNA gene sequences showed that HHB‐1 is classified within the *Magnetococcia* class (corresponding to the previous *Ca*. Etaproteobacteria class) of the *Pseudomonadota* (formerly *Proteobacteria*) phylum (Figure 3).

**FIGURE 4:**
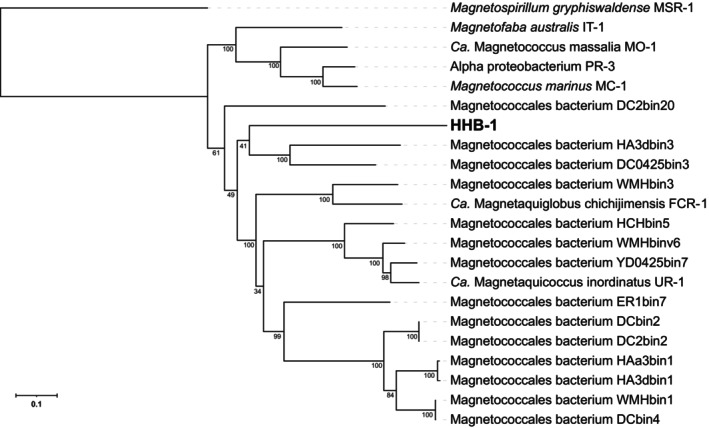
Maximum‐likelihood phylogenomic tree showing the placement of strain HHB‐1 within the class *Ca*. Etaproteobacteria, branch support values calculated from 1000 ultrafast bootstrap replicates. *Magnetospirillum gryphiswaldense* MSR‐1 used as outgroups to root the tree. The scale bar represents amino acid substitutions per site.

For further genome phylogenetic analysis, a concatenated alignment of 120 bacterial single‐copy proteins was used for maximum‐likelihood tree reconstruction using GTDB‐Tk. All the magnetotactic cocci formed a clade. Three cultivated magnetotactic cocci, including IT‐1, MO‐1, MC‐1 and PR‐3 grouped into a single branch (bootstrap value = 100%), while HHB‐1, along with *Ca*. Mac. inordinatus UR‐1, *Ca*. Mag. chichijimensis FCR‐1, and some other magnetotactic cocci MAGs formed another independent and well‐supported clade within the order *Magnetococcales* (bootstrap value = 61%) (Figure 4). However, HHB‐1 does not cluster with any previous genome in the clade and clustered with two magnetotactic cocci MAGs with a lower support (bootstrap value = 41%), indicating HHB‐1 is clearly distinct from the candidate family “*Ca*. Magnetaquicoccaceae,” represented by FCR‐1 and UR‐1 (Koziaeva et al. 2019; Shimoshige et al. 2025). This distinct phylogenetic placement indicates the unique evolutionary position of HHB‐1. Given its distant relationship to the above lineages, coupled with the 16S rRNA gene divergence, HHB‐1 may represent a novel evolutionary branch within *Magnetococcales*.

**FIGURE 5:**
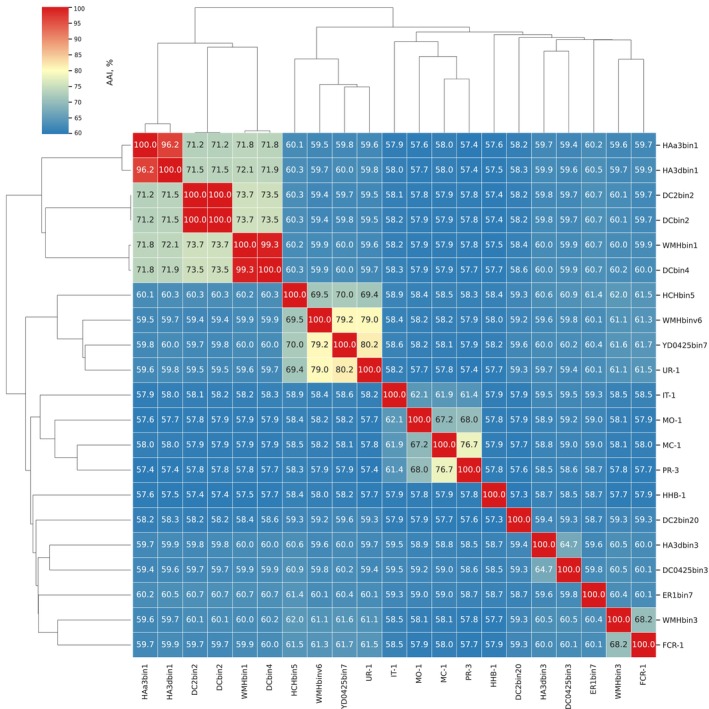
AAI‐based whole‐genome comparisons among Magnetococcales strains are shown in the heatmap. The colour gradient in the top‐left corner indicates similarity levels.

To evaluate the genomic relatedness of HHB‐1 to other members of the order *Magnetococcales*, pairwise average AAI values were calculated based on representative genomes and MAGs (Figure 5). HHB‐1 exhibited AAI values ranging from 57.3% to 58.7% across all *Magnetococcales* genomes analysed, which is well below the commonly accepted genus‐level threshold (typically ≥ 65%), indicating low genomic similarity and distant relatedness to other members of the order (Konstantinidis et al. 2017). Comparisons with the cultivated magnetotactic cocci IT‐1, MO‐1 and MC‐1 yielded AAI values between 57.8% and 57.9%. HHB‐1 shared AAI values of 57.7% with UR‐1 and 57.9% with FCR‐1, both proposed members of the candidate family Magnetaquicoccaceae. These AAI values fall within the family‐level threshold (typically 45%–65%) (Konstantinidis et al. 2017) and specifically correspond to the proposed 55%–56% lower boundary for delineating families within *Magnetococcales*, which has been shown to align well with phylogenomic branching patterns (Koziaeva et al. 2019), suggesting that HHB‐1 likely represents a distinct lineage. HHB‐1 does not cluster with any other genome or MAG within this clade based on AAI heatmap and hierarchical clustering, further supporting its unique phylogenetic status. Taken together, the AAI‐based comparative analysis corroborates the phylogenomic inference and supports the classification of HHB‐1 as a representative of a novel candidate genus within a distinct family, representing a deeply divergent lineage in the order Magnetococcales. Herein, we propose the designation “*Ca*. Magnetomacrococcaceae” for the novel family and “*Ca*. Magnetomacrococcus sanyaensis” for strain HHB‐1.

### 3.6 Iron Metabolism and Magnetosome Biomineralization

A role for iron metabolism in the physiology of HHB‐1 is implied by genetic analysis, particularly in magnetosome synthesis, respiratory chain function and energy production. The genome of HHB‐1 encoded genes for the ferrous iron transporter *FeoB*, which likely mediates Fe^2+^ uptake under anaerobic or microaerobic conditions (Cartron et al. 2006), and *TonB*, which generally functions to energize outer membrane TonB‐dependent receptors (Noinaj et al. 2010). While no TonB‐dependent receptors directly related to siderophore–ferric iron uptake were identified in the current genome assembly.

The MGC of strain HHB‐1 exhibits a genomic structure and gene composition highly similar to those of other magnetotactic cocci. Magnetosome‐associated genes in HHB‐1 are distributed across two genomic contigs and include a total of 17 *mam* (magnetosome membrane) genes and one *mms* (magnetic particle‐membrane specific) gene, encompassing all known core *mam* components along with a subset of accessory genes (Taoka et al. 2023). The HHB‐1 MGC encodes the mamAB operon core genes, such as *mamA*, *mamB*, *mamE*, *mamF‐like*, *mamM*, *mamO*, *mamP* and *mamQ*, as well as several accessory genes including *mamD*, *mamX*, and *mamZ* from the *mamXY* operon, and *mmsF* from the *mms6* operon. The gene *maq1* was previously considered specific to the MGCs of the Ca. Magnetaquicoccaceae family (Koziaeva et al. 2019; Shimoshige et al. 2025). Here, however, we have detected a homologue of *maq1* within the MGC of HHB‐1, a putative member of the novel family Ca. Magnetomacrococcaceae. This homologue shares limited amino acid sequence similarity (33.3% to FCR‐1 and 37.5% to UR‐1; Figure S1), suggesting it may represent a distinct group within the Maq1 protein. These genes suggest that HHB‐1 harbours a nearly complete and functionally conserved MGC, reflecting its capacity for magnetosome biomineralization.

The HHB‐1 genome encodes key magnetosome‐associated proteins (MAPs) involved in redox regulation and iron handling. For example, MamP harbours two MTB‐unique heme‐binding c‐type cytochrome domains, termed magnetochromes, which endow the protein with iron oxidase activity (Siponen et al. 2013; Siponen et al. 2012). In *Paramagnetospirillum* (formerly *Magnetospirillum*) *magneticum* AMB‐1, MamP has been experimentally demonstrated to catalyse Fe(II) oxidation during magnetite biomineralization (Jones et al. 2015; Taoka et al. 2023; Amor et al. 2024). MamB and MamM are members of the cation diffusion facilitator (CDF) family, whose general function is to transport divalent transition metal cations, such as Zn^2+^, Co^2+^, Cd^2+^, Mn^2+^, Ni^2+^ and Fe^2+^, from the cytoplasm into intracellular compartments or to the periplasmic/extracellular space via the proton motive force (Paulsen and Saier Jr 1997; Barber‐Zucker et al. 2016). Their specific role has been experimentally validated in *Magnetospirillum gryphiswaldense* MSR‐1, showing that they mediate iron transport into magnetosomes during biomineralization (Uebe et al. 2011).

**FIGURE 6:**
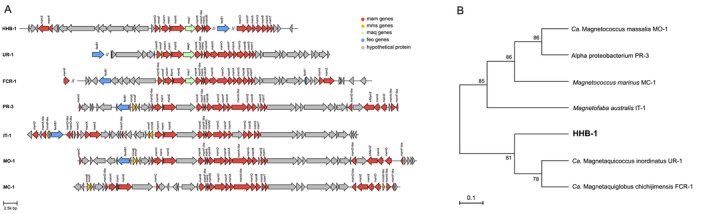
MGC region organization and phylogeny based on magnetosome proteins. (A) Organization of the MGC regions in the genomes of *Magnetococcales* strains. Arrows indicate gene content and transcriptional direction. UR‐1: *Ca*. Magnetaquicoccus inordinatus UR‐1; FCR‐1: *Ca*. Magnetaquiglobus chichijimensis FCR‐1; PR‐3: Alphaproteobacterium PR‐3; IT‐1: *Magnetofaba australis* IT‐1; MO‐1: *Ca*. Magnetococcus massalia MO‐1; MC‐1: *Magnetococcus marinus* MC‐1. (B) Maximum‐likelihood (ML) phylogenetic tree constructed based on the concatenated sequences of 14 core magnetosome proteins (MamH, I, E, K, F‐like, L, M, O, P, A, Q, B, S and T), illustrating the evolutionary relationships between strain HHB‐1 and the six *Magnetococcales* strains. The scale bar indicates the number of amino acid substitutions per site.

Comparative analysis of MGC among HHB‐1 and six *Magnetococcales* strains was conducted using the Clinker module in MagCluster (Ji et al. 2022). Pairwise comparisons revealed variable levels of sequence conservation (Table S2). Comparisons with UR‐1 showed higher identity (37%–67%) and similarity (49%–80%) for some proteins. Despite these sequence variations, all strains exhibited a conserved gene order and overall MGC organization, with most magnetosome genes maintaining a consistent linear arrangement (Figure 6A).

To explore the evolutionary relationships of core magnetosome proteins, a maximum‐likelihood phylogenetic tree was constructed based on the concatenated amino acid sequences of 14 conserved magnetosome proteins (MamH, I, E, K, F‐like, L, M, O, P, A, Q, B, S and T) (Figure 6B). The resulting tree clearly resolved two major clades within the order *Magnetococcales*. One clade comprised the cultivated magnetotactic cocci *Ca*. Mc. massalia MO‐1, PR‐3, *Mc. marinus* MC‐1 and *Mf. australis* IT‐1, which formed a well‐supported cluster (bootstrap values 85–86). The other clade included HHB‐1, *Ca*. Mac. inordinatus UR‐1 and *Ca*. Mag. chichijimensis FCR‐1. HHB‐1 was placed on a distinct branch with a bootstrap support of 81, clearly separated from the UR‐1 and FCR‐1 lineage (bootstrap = 78), suggesting that HHB‐1 represents a novel phylogenetic lineage with a unique set of core magnetosome proteins.

Comparative analysis of the MGC revealed genomic plasticity in HHB‐1. Mobile element insertions were observed flanking the mamXY operon, indicating possible horizontal gene transfer or genome rearrangements. The *mmsF* locus exhibited lineage‐specific duplication events, suggesting gene expansion within certain evolutionary clades. Furthermore, strain‐specific insertion sequences (IS elements) were found in the intergenic region between *mamA* and *mamB*, potentially affecting gene regulation and operon integrity.

## 4 Discussion

Our study revealed a novel predominant magnetotactic cocci strain HHB‐1 within intertidal zone sediments of Houhai Bay, located in the South China Sea. Phylogenetically, the HHB‐1 could be classified into a novel family of *Magnetococcia* class according to the results of 16S rRNA gene, MAG and AAI analysis. In contrast to MO‐1, MC‐1, IT‐1, FCR‐1 and UR‐1, HHB‐1 exhibited marked differences (Table 1). The HHB‐1 cells exhibited a swimming speed of 39.48 ± 10.80 μm/s under an external magnetic field of ~25.5Gs (~50× Earth's magnetic field). Although direct comparisons should be interpreted with caution due to the different magnetic field conditions used across studies, the swimming speed of HHB‐1 appears slower than those reported for the three axenically cultured magnetotactic cocci (MO‐1, MC‐1 and IT‐1), all of which produce a single chain of less than to a dozen magnetite crystals (Lefèvre et al. 2009; Bazylinski et al. 2013; Morillo et al. 2014). Specifically, MO‐1 was measured under a parallel magnetic field of approximately 5 times the Earth's field (personal communication Dr. Long‐Fei Wu) and typically exhibits speeds of 100~300 μm/s, with freshly harvested cells reaching up to 350 μm/s (Lefèvre et al. 2009). In the case of MC‐1, the mean swimming speed in fresh culture was 197 ± 15 μm/s. However, the external field strength was not specified in the original report (Bazylinski et al. 2013). IT‐1 displayed an average swimming speed of 186 ± 63 μm/s, with a maximum of 300 μm/s, measured under the magnetic field of a bar magnet (Morillo et al. 2014). Additionally, the motility speed of HHB‐1 cells was also slightly slower than that of the axenic culture of FCR‐1 (58.2 ± 9.8 μm/s), which contains unchained magnetosomes and were tested under a bar magnet generating ~80 mT (~160× Earth's field). Thus, even under a stronger external magnetic field than that applied to MO‐1, HHB‐1 cells swam much more slowly than the single‐chain magnetosome cocci (MO‐1). In contrast, although the swimming speed of HHB‐1 was similar to that of FCR‐1, the latter was tested under a magnetic field more than three times stronger than that applied to HHB‐1. These observations indicate that HHB‐1 exhibits intrinsically lower motility compared with typical single‐chain magnetosome magnetotactic cocci. Theoretically, the multi‐chain magnetosome configuration observed in HHB‐1 cells may adopt an antiparallel arrangement as a result of energy minimization principles, which represents a stable configuration. This antiparallel coupling among the chains can reduce the net magnetic dipole moment, thereby weakening the overall magnetotactic efficiency of the cells. By contrast, a single linear chain of magnetosomes maximizes the magnetic dipole moment of the cell, enhancing its response to geomagnetic fields, whereas dispersed arrangements may diminish this polarization effect due to less effective vector alignment (Shimoshige et al. 2025). Taken together, both multi‐chain and dispersed configurations can result in a reduced net magnetic dipole moment, which may explain the relatively slower swimming velocity observed in HHB‐1 and FCR‐1 under an applied magnetic field.

**TABLE 1:** Characterization comparison among different magnetococci strains.

Strain names	MC‐1	MO‐1	IT‐1	UR‐1	FCR‐1	HHB‐1
Cell size (μm)	1~2	1.3~1.9	1.1~1.4	1.2~1.5	1.3~1.9	2.9~3.7
Swimming speed (μm/s)^a^	197 ± 15	~300	186 ± 63	ND	58.2 ± 9.8	39.5 ± 10.8
MS chain number	1	1	1	Cluster	Cluster	Multi‐chain
MS number	10 ± 2	17 ± 5	10 ± 3	18~38	14 ± 4	91 ± 17
MS shape	Pseudo‐hexagonal prism	Elongated cubooctahedra	Octahedra	Elongated hexagonal prism	Truncated hexagonal prism	Prism
MS size (length × width, nm)	72 ± 11 × 70 ± 13	64 ± 20 × 57 ± 17	83 ± 26 × 74 ± 24	77 ± 12 × 48 ± 8	77 ± 14 × 50 ± 10	104 ± 28 × 62 ± 18
MS composition	Fe_3_O_4_	Fe_3_O_4_	Fe_3_O_4_	Fe_3_O_4_	Fe_3_O_4_	Fe_3_O_4_
Inclusions	polyP	polyP Lipid globules	polyP Sulphur granules	polyP	Ca‐polyP Mg‐polyP	Ca‐polyP Mg‐polyP
Natural habitat	Marine water	Marine intertidal sediment	Marine coastal lagoon sediment	Freshwater river sediment	Freshwater dam sediment	Marine intertidal sediment
Cultivation status	Pure culture	Pure culture	Pure culture	Uncultured	Pure culture	Uncultured
References	(Bazylinski et al. 2013)	(Lefèvre et al. 2009)	(Morillo et al. 2014)	(Koziaeva et al. 2019)	(Shimoshige et al. 2025)	This study

Abbreviations: MS, magnetosome; ND, not detected; polyP, polyphosphate.

^a^ Swimming speeds were measured under different external magnetic field conditions and are therefore not directly comparable. HHB‐1 was tested under ~25.5 Gs (≈50× Earth's magnetic field), MO‐1 under a parallel magnetic field generated by a custom‐made coil (~5× Earth's field), MC‐1 without reporting the field strength, IT‐1 under the magnetic field of a bar magnet (not quantified) and FCR‐1 under a bar magnet generating ~80 mT (≈160× Earth's field).

Typically, magnetotactic cocci exhibit cell sizes within the range of 1~2.5 μm (Liu, Liu, Zhao, et al. 2021). However, HHB‐1 displayed cellular dimensions substantially exceeding those of common strains (Table 1). The enlarged volume may accommodate specialized organelles, such as multi‐magnetosome chains or Ca‐Mg‐polyP granules, requiring additional cytoplasmic space. As is well known, bacterial polyP granules serve as the primary site for the sequestration of metals, such as Ca, Mg, Na, K and other cations. TEM and EDX analysis revealed Ca‐Mg‐polyP inclusions within the HHB‐1 cells, which also have been found in *Ca*. Azospirillum magnetospirillum WYHS‐1 (Li et al. 2021) and *Ca*. Mag. chichijimensis FCR‐1 (Shimoshige et al. 2025). Intracellular polyP granules appear to be ubiquitously distributed as conserved storage structures across phylogenetically diverse MTB, with particular predominance in magnetococci (Keim et al. 2005; Lefèvre et al. 2009; Bazylinski et al. 2013; Morillo et al. 2014; Koziaeva et al. 2019; Schulz‐Vogt et al. 2019; Liu, Liu, Ren, et al. 2021; Bidaud et al. 2022; Shimoshige et al. 2025). Previous studies believed that polyP within many bacterial cells serve a variety of crucial functions, including energy storage, regulation of cellular processes and protection against oxidative stress (Hupfer et al. 2007; Gray and Jakob 2015; Akbari et al. 2021). Therefore, the presence of polyP granules may enhance the ability of HHB‐1 to survive and thrive in challenging environmental conditions. This may also have allowed HHB‐1 to dominate the sampled sediment. In stratified aquatic environments, certain magnetotactic cocci have been shown to accumulate giant polyP inclusions occupying up to 90% of the cellular volume under anoxic conditions (Bidaud et al. 2022). This suggests that they may mediate localized phosphorus enrichment and redistribution through a “polyP accumulation—release” mechanism. It also implies that these magnetotactic cocci may possess the capacity to efficiently synthesize and regulate polyP under oxygen‐depleted conditions, thereby playing an important role in P cycling. In this study, dumbbell‐shaped Ca‐Mg‐polyP granules under TEM were presumed to have been released from the HHB‐1 cells under osmotic stress or other unknown stress conditions encountered during sample preparation. By counting the number of dumbbell‐shaped granules relative to HHB‐1 cells in the TEM images, we estimated a ratio of approximately 9:10, suggesting that the proportion of HHB‐1 cells containing Ca‐Mg‐polyP granules in our sample was not less than 90%. The dimensions of these Ca‐Mg‐polyP granules were 2.87 ± 0.4 μm in length and 2.0 ± 0.3 μm in width (*n* = 10). TEM observations showed that HHB‐1 cells measured 3.8 ± 0.4 μm × 2.7 ± 0.3 μm. Assuming both the granules and the cells are approximately rotational ellipsoidal, the granules are estimated to occupy ~41.4% of the cell volume, suggesting that HHB‐1 may contribute to the P cycle of intertidal zone sediments.

The multi‐chain arrangement of magnetosomes in HHB‐1 is significantly different from the single‐chain magnetosomes typically observed in cultivated MTB, such as MO‐1, MC‐1 and IT‐1 (Lefèvre et al. 2009; Bazylinski et al. 2013; Morillo et al. 2014). In the phylum *Pseudomonadota*, the configuration of magnetosome chains appears to be associated with the copy number and sequence similarity of the *mamK* gene. Among *Ca*. Etaproteobacteria, magnetotactic bacterial strains with multiple copies of the *mamK* gene and relatively low similarity between the *mamK* sequences (such as protein sequence similarity < ~67%) appear to assemble twisted or partial chains or dispersed aggregates (Liu et al. 2022). However, HHB‐1 appears to harbour only a single *mamK* copy while still forming multiple magnetosome chains. This observation may contradict the hypothesis that *mamK* copy number and/or sequence similarity alone determine chain morphology. Nevertheless, we cannot entirely rule out the possibility that additional *mamK* copies are absent due to genome incompleteness. Taken together, our results suggest that other regulatory or structural mechanisms, in addition to *mamK*, likely contribute to the atypical magnetosome organization in HHB‐1. The gene *maq1* has been identified exclusively in members of the family *Ca*. Magnetaquicoccaceae, such as FCR‐1 and UR‐1, both of which exhibit non‐linear or unchained magnetosome arrangements (Koziaeva et al. 2019; Shimoshige et al. 2025). This observation raises a plausible hypothesis that *maq1* may play a potential role in regulating magnetosome alignment or crystal organization independently of the MamK‐mediated cytoskeletal control system (Shimoshige et al. 2025). In this study, HHB‐1, which assembles multi‐chain magnetosomes and may represent a new family (*Ca*. Magnetomacrococcaceae), also contains homologues of *maq1*. Therefore, the presence of *maq1* does not provide evidence for involvement in magnetosome chain formation, and other genes may contribute to controlling the number of magnetosome chains in HHB‐1. Within the class *Magnetococcia*, the *mamC* gene shows a distribution pattern restricted to strains (e.g., MO‐1, MC‐1, IT‐1, PR‐3) that synthesize single‐chain magnetosomes, while it is absent from strains with unchained magnetosomes (e.g., UR‐1 and FCR‐1) as well as the multi‐chain magnetosome‐forming strain HHB‐1 identified in this study (Figure 6A). Previous in vitro studies have demonstrated that the *mamC* gene from strain MC‐1 contributes to controlling magnetite crystal size (Valverde‐Tercedor et al. 2015). However, the lack of a genetic manipulation system for magnetotactic cocci still hampers verification of its in vivo function and further exploration of its regulatory mechanisms.

## 5 Conclusion

In this study, we characterized a novel marine magnetotactic coccus, HHB‐1, isolated from intertidal sediments. HHB‐1 exhibits distinctive morphological features, including large cell size, multiple magnetosome chains and prominent intracellular Ca/Mg‐rich polyP granules. Its MGC is nearly complete and structurally conserved. Genomic and phylogenetic analyses place HHB‐1 within a deeply branching, distinct lineage of *Magnetococcales*, separate from known strains such as MC‐1, MO‐1, IT‐1, UR‐1 and FCR‐1. These findings expand our understanding of magnetotactic bacterial diversity and offer new perspectives on their biomineralization mechanisms and evolutionary adaptation in marine environments.

## Author Contributions

**Yuzan Che:** investigation, validation, formal analysis, writing – original draft, writing – review and editing, data curation. **Wenyan Zhang:** writing – original draft, writing – review and editing, visualization, data curation, formal analysis, validation. **Yi Dong:** investigation, formal analysis, writing – review and editing. **Min Liu:** investigation, resources, writing – review and editing. **Tian Xiao:** writing – review and editing, formal analysis, conceptualization. **Jin‐Yong Zhang:** supervision, writing – review and editing. **Hongmiao Pan:** conceptualization, funding acquisition, writing – original draft, writing – review and editing, project administration, supervision, formal analysis.

## Funding

This work was supported by the National Natural Science Foundation of China, 42176123.

## Conflicts of Interest

The authors declare no conflicts of interest.

## Supporting information


**Data S1:** Supporting Information.

## Data Availability

Publicly available datasets were analysed in this study. These data can be found here: The original genome sequence of *Ca*. Magnetomacrococcus sanyaensis HHB‐1 has been deposited in GenBank under the accession number JBQGBW000000000.
