# Supplementary material for: Characterization and Phylogenetic Analysis of a Novel Uncultivated Magnetotactic Coccus Harbouring Multi‐Chain Magnetosomes
Source: Environ Microbiol Rep. 2026 Jan 11;18(1):e70266. doi: 10.1111/1758-2229.70266 (PMC12791574; doi:10.1111/1758-2229.70266)
Supplement: Supplementary file 1 — Data S1: Supporting Information. [file EMI4-18-e70266-s001.docx]

**Supplementary:**

Table S1 General genomic features of the draft genome of HHB-1

| Parameter | HHB-1 |
| --- | --- |
| Total genome size (Mb) | 4.96 |
| N50 (bp) | 28302 |
| Number of contigs | 444 |
| Number of coding sequences (CDS)  Average contig length (bp)  5/16/23S rRNA  tRNA  GC content  Completeness  Contamination | 3981  11172.55  1/1/0  48  46.46%  94.07%  7.14% |

Table S2 Sequence Identity and Gene Similarity Between HHB-1 and Magnetococcales Strains

| Strain | Sequence Identity (%) | Avg. Identity ± SD | Gene Similarity (%) | Avg. Similarity ± SD |
| --- | --- | --- | --- | --- |
| UR-1 | 38.00 – 67.00 | 52.00 ± 8.62 | 49.00 – 80.00 | 65.69 ± 9.70 |
| FCR-1 | 33.00 – 64.00 | 49.43 ± 9.13 | 43.00 – 76.00 | 63.21 ± 10.77 |
| PR-3 | 38.00 – 60.00 | 47.93 ± 6.80 | 50.00 – 79.00 | 62.86 ± 8.35 |
| IT-1 | 34.00 – 60.00 | 47.64 ± 7.15 | 47.00 – 77.00 | 62.21 ± 8.81 |
| MC-1 | 38.00 – 58.00 | 47.29 ± 7.10 | 49.00 – 78.00 | 62.71 ± 8.84 |
| MO-1 | 38.00 – 58.00 | 46.00 ± 5.48 | 50.00 – 72.00 | 60.79 ± 6.29 |


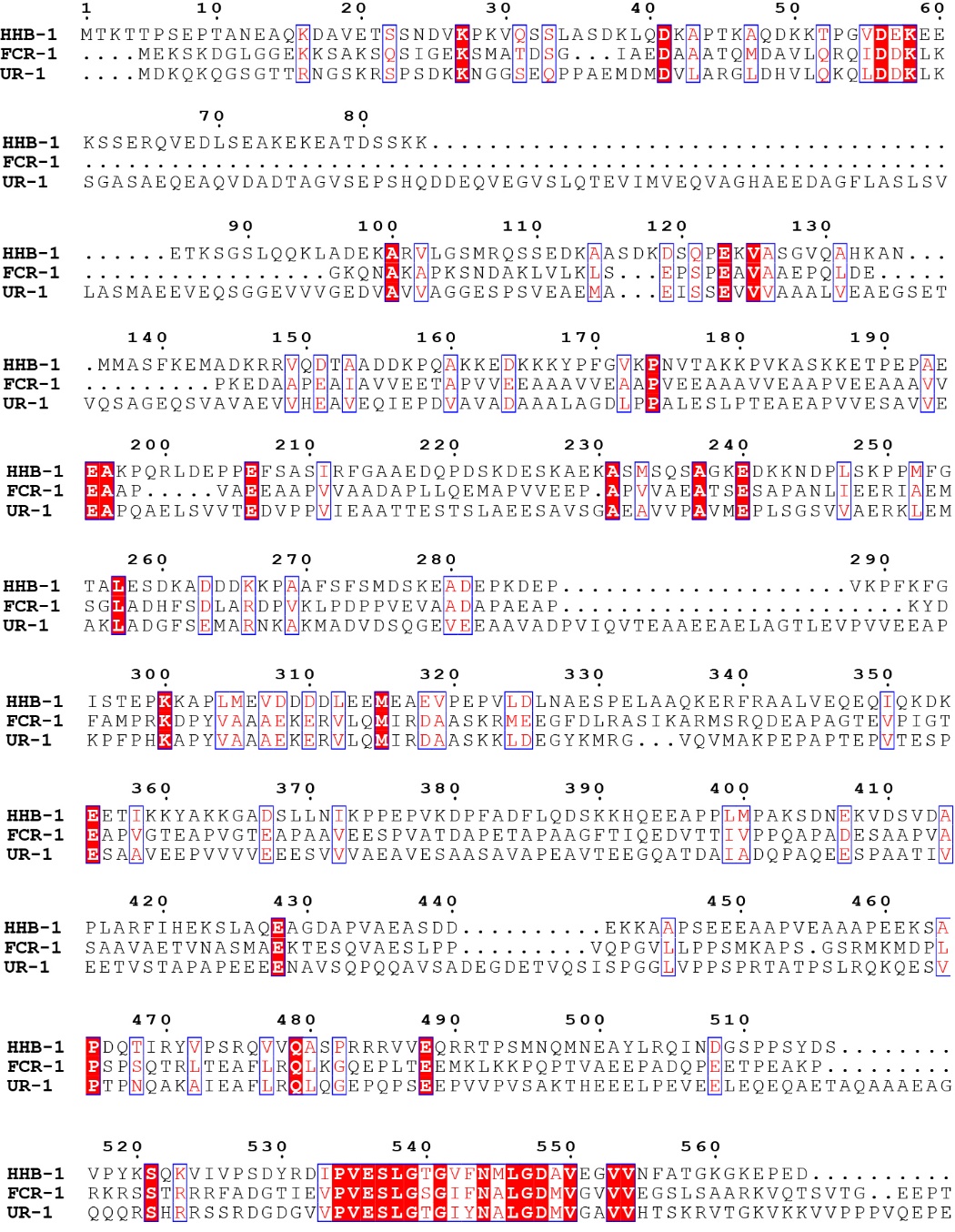


Fig S1. Amino acid sequences alignment of the *maq1* gene among the three strains
